# The Two-Dimensional Nanocomposite of Molybdenum Disulfide and Nitrogen-Doped Graphene Oxide for Efficient Counter Electrode of Dye-Sensitized Solar Cells

**DOI:** 10.1186/s11671-016-1277-0

**Published:** 2016-02-29

**Authors:** Chao-Kuang Cheng, Che-Hsien Lin, Hsuan-Chung Wu, Chen-Chi M. Ma, Tsung-Kuang Yeh, Huei-Yu Chou, Chuen-Horng Tsai, Chien-Kuo Hsieh

**Affiliations:** Department of Materials Engineering, Ming Chi University of Technology, New Taipei City, 24301 Taiwan, Republic of China; Department of Engineering and System Science, National Tsing Hua University, Hsinchu, 30013 Taiwan, Republic of China; Department of Chemical Engineering, National Tsing Hua University, Hsinchu, 30013 Taiwan, Republic of China

**Keywords:** Molybdenum disulfide, Nitrogen-doped, Graphene oxide, Counter electrode, Dye-sensitized solar cells

## Abstract

In this study, we reported the synthesis of the two-dimensional (2D) nanocomposite of molybdenum disulfide and nitrogen-doped graphene oxide (MoS_2_/nGO) as a platinum-free counter electrode (CE) for dye-sensitized solar cells (DSSCs). X-ray photoelectron spectroscopy (XPS), high-resolution transmission electron microscopy (HRTEM), and Raman spectroscopy were used to examine the characteristics of the 2D nanocomposite of MoS_2_/nGO. The cyclic voltammetry (CV), electrochemical impedance spectra (EIS), and the Tafel polarization measurements were carried out to examine the electrocatalytic abilities. XPS and Raman results showed the 2D behaviors of the prepared nanomaterials. HRTEM micrographs showed the direct evidence of the 2D nanocomposite of MoS_2_/nGO. The results of electrocatalytic examinations indicated the MoS_2_/nGO owning the low charge transfer resistance, high electrocatalytic activity, and fast reaction kinetics for the reduction of triiodide to iodide on the electrolyte–electrode interface. The 2D nanocomposite of MoS_2_/nGO combined the advantages of the high specific surface of nGO and the plenty edge sites of MoS_2_ and showed the promoted properties different from those of their individual constituents to create a new outstanding property. The DSSC with MoS_2_/nGO nanocomposite CE showed a photovoltaic conversion efficiency (PCE) of 5.95 % under an illumination of AM 1.5 (100 mW/cm^2^), which was up to 92.2 % of the DSSC with the conventional platinum (Pt) CE (PCE = 6.43 %). These results reveal the potential of the MoS_2_/nGO nanocomposite in the use of low-cost, scalable, and efficient Pt-free CEs for DSSCs.

## Background

Dye-sensitized solar cells (DSSCs) are considered as the next-generation solar cells, because of low cost, easy process, and low energy consumption [1–4]. The conventional DSSC is composed of a ruthenium dye-sensitized titanium dioxide (TiO_2_) working electrode (WE), an electrolyte containing iodine ions, and a platinum (Pt) catalyst counter electrode (CE) [1]. In order to ensure the performance of DSSCs, Pt plays an important catalytic material for DSSCs. However, due to Pt as an expensive and scarce material, it is necessary to find an alternative economical material to replace Pt for profitable DSSCs.

Carbon materials show the advantages of their low cost, high surface area, and high electric conductivity. Recently, low-dimensional carbon nanomaterials, such as carbon nanotubes (CNTs) [5, 6], carbon nanofibers (CNFs) [7, 8], graphene [9–11], and graphene oxide (GO) [9, 12], have been found to replace Pt for DSSCs. Some recent reports have claimed that the performance of carbon nanomaterials in the CE of DSSCs benefited from the presence of the plenty active sites, which may be attributed to the defect sites [7, 11, 12]. GO is an important carbon nanomaterial in the graphene family. In addition, due to their unique two-dimensional (2D) nanostructural feature, high specific surface area, electrochemical stability, and hydrophilic oxygen-containing groups, GO have been widely used as anchored templates to synthesize nanocomposites for DSSC CEs [13, 14]. However, GO suffered relatively high oxygen-containing defects and structural defects such as vacancies and topological defects on the surface. The plenty oxygen-containing defects on the GO surface brought out the low exchange current density, because the surface defects cut down the electrical conductivity [15]. In order to overcome the disadvantages of GO, nitrogen atoms doped into GO to synthesize the nitrogen-doped GO (nGO) were demonstrated to repair the defects, which provided the improvement in the electronic structure of GO [16]. On the other hand, the nitrogen-doped process broadens the electrochemical application area of a variety of carbon-based nanomaterials, including the nitrogen-doped CNT for glucose sensor [17], the nitrogen-doped reduced graphene oxide (N-rGO) for the DSSC [18], the nitrogen-doped graphene, and the N-rGO for supercapacitors [19, 20]. Nitrogen-doped carbon-based nanomaterials not only can adjust the work function of graphene [21] but also can improve the electrical conductivity and the electrochemical properties of the graphene family.

Recently, stimulated by the discovery of the 2D nanomaterial graphene, the transition metal dichalcogenides (TMDCs) with the 2D nanostructure have attracted considerable attention [22–28]. Molybdenum disulfide (MoS_2_) is a typical TMDC, one unit sheet pile up with three layers which is S–Mo–S arranged sequentially [25, 26]. MoS_2_ has been found recently with its excellent catalytic activity because their edge planes provided abundant active sites for catalytic reactions [24, 29].

In this study, we synthesized the 2D nanocomposite of molybdenum disulfide and nitrogen-doped graphene oxide (MoS_2_/nGO) based on the 2D nanomaterials of MoS_2_ and nGO. Due to the high specific surface of nGO, the nGO was used as the anchored templates to synthesize the MoS_2_ on the surface as the 2D nanocomposite of MoS_2_/nGO. The N-doping atoms of nGO not only repaired the vacancies but also replaced the O atoms of GO to enhance the electrical conductivity. In combining the advantages of the high specific surface of nGO with the improving conductivity and the excellent catalytic activity of MoS_2_, the 2D nanocomposite of MoS_2_/nGO showed the outstanding electrocatalytic activities. The synthesized MoS_2_/nGO nanocomposite was used as a CE to assemble into a Pt-free DSSC and examined under the illumination of AM 1.5 (100 mW/cm^2^). The DSSC with MoS_2_/nGO nanocomposite CE exhibited the impressive photovoltaic conversion efficiency (PCE, *η*) of 5.95 %. It was up to 92.2 % compared with the DSSC using the conventional Pt CE (*η* = 6.43 %). The results showed that MoS_2_/nGO nanocomposites have great potential for DSSC-related applications and indicated its potential as an alternative to replace Pt.

## Methods

### Synthesis of MoS_2_/nGO Nanocomposite CE

Three steps were used for preparing the MoS_2_/nGO nanocomposite CE. In step 1, GO nanosheets were synthesized from natural graphite flakes (Alfa Aesar, Ultra Superior Purity >99.9999 %) by using modified Hummer’s method [30]. In step 2, hydrothermal synthesis method was used to dope nitrogen atoms into GO to obtain nGO nanosheets [16], 120 mg GO dissolved in 120 mL deionized water, and followed by 1 h of sonication. The as-synthesized GO solution was prepared by using 3 mL ammonia (NH_4_OH) and 2 mL hydrazine hydrate (N_2_H_4_) as the reducing agents. Subsequently, the aforementioned solution was transferred into a Teflon-lined autoclave and heated to 120 °C for 3 h to synthesize the nGO. After hydrothermal synthesis reaction, the nGO precipitates were washed with deionized water for several times and collected by centrifugation and then dried in vacuum. In step 3, 30 mg ammonium tetrathiomolybdate ((NH_4_)_2_MoS_4_) powder (ProChem, Inc., purity of 99.99 %) and 30 mg nGO powder were added to 4 mL *N*,*N*-dimethylformamide (DMF) for dispersion and then sonicated for 1 day. Subsequently, the dispersed solution was coated on fluorine-doped tin oxide (FTO) glass substrates (TEC-7, 2.2 mm, Hartford) by spin coating technology. Then, the obtained sample was dried in air for 1 h. Finally, the prepared sample was heated in the gas mixture (H_2_/Ar = 1:9) at 300 °C for 30 min by a typical homemade hot-wall thermal chemical vapor deposition (CVD) system (a horizontal furnace and a quartz tube) to obtain the 2D nanocomposite of MoS_2_/nGO CE.

### Preparation of nGO, MoS_2_, and Pt CEs

For preparing the nGO CE, 30 mg of nGO powder was added to 3 mL of DMF dispersion and sonicated for 1 day. Subsequently, the nGO solution was spin-coated on FTO glass substrates and dried in vacuum at 120 °C for 30 min in our homemade hot-wall thermal CVD system. The MoS_2_ CE was prepared by using 4 wt% dispersed solution (0.8 g (NH_4_)_2_MoS_4_ powder in 20 mL DMF dispersion). The solution was coated on FTO glass substrates and dried in air for 1 h. Finally, the sample was heated in the gas mixture (H_2_/Ar = 1:9) at 300 °C for 30 min in the thermal CVD system to obtain MoS_2_ CE. For preparing the reference Pt CE, 2 mM H_2_PtCl_6_ isopropanol solution is coated on FTO glass substrates and heated to 450 °C for 20 min by thermal-reduced method [31].

### Fabrication of DSSCs

For the preparation of the WE, nanocrystalline TiO_2_ was coated on FTO glass substrates by using screen print technology. The coated TiO_2_ samples were then heated to 550 °C for 30 min in the air. After the sinter process, the WE-coated TiO_2_ on FTO glass substrates was immersed into N719 (Solaronix) solution (0.3 mM in a mixture of acetonitrile and tertbutylalcohol (volume ratio 1:1)) at 50 °C for 1 h. Subsequently, the dye-adsorbed TiO_2_ WE was washed with acetonitrile for a few seconds to remove the remaining dye and dried at room temperature. Finally, the DSSCs were consisted of the WE, various CEs, and the iodide-based electrolyte (AN-50, Solaronix) with the 60-μm-thick hot-melt spacer (SX1170-60, Solaronix) between the two electrodes.

### Characterizations

X-ray photoelectron spectroscopy (XPS) (PHI Quantera SXM/AES 650 Auger Electron Spectrometer (ULVAC-PHI INC., Japan) equipped with a hemispherical electron analyzer and a scanning monochromated Al K-α (hv = 1486.6 eV) X-ray source) was used to examine the chemical states of the prepared samples. Raman spectroscopy was performed with a confocal micro-Raman spectroscope (LABRAM HR 800 UV, Japan) using a 632.8-nm laser source (50 m W) with a spot size of approximately 1 μm to characterize the prepared CEs. The nanostructures of nGO nanosheet and MoS_2_/nGO nanocomposite were investigated by using the high-resolution transmission electron microscopy (HRTEM, JEOL-2100F, Japan). Cyclic voltammetry (CV) measurements were carried out by using a potentiostat/galvanostat (PGSTAT 302N, Autolab, Eco Chemie, Netherlands) in a three-electrode configuration to examine the electrocatalytic activities of our prepared CEs. The Pt wire and an Ag/AgNO_3_ electrode were used as the counter and reference electrodes for the CV measurements, respectively. The solution used for CV measurements contained 1 mM I_2_, 10 mM LiI, and 0.1 M LiClO_4_ in acetonitrile [7]. Electrochemical impedance spectra (EIS) were obtained by using the aforementioned potentiostat/galvanostat equipped with a frequency response analysis (FRA) module. The Nyquist plots were scanned from 10^6^ to 10^−2^ Hz, and an applied voltage of 10 mV was used. The EIS results were fitted by using an equivalent circuit model with Autolab FRA software (v4.9, EcoChemie B.V.). The Tafel polarization measurements were also measured by the same potentiostat/galvanostat equipped with a linear polarization module. Both EIS and the Tafel polarization measurements were obtained by using symmetrical devices based on two identical CEs in the dark. All photocurrent density–voltage measurements of DSSCs were measured under the simulated solar illumination (AM 1.5, 100 mW/cm^2^, Oriel 91160, Newport Corporation, USA), which was equipped with an AM 1.5G filter (Oriel 81088A, Newport Corporation, USA) and a 300-W xenon lamp (Oriel 6258, Newport Corporation, USA). The intensity of the simulated incident light was calibrated using a reference Si cell (calibrated at NREL, PVM-81).

## Results and Discussion

### Composition and Structural Features

**Fig. 1:**
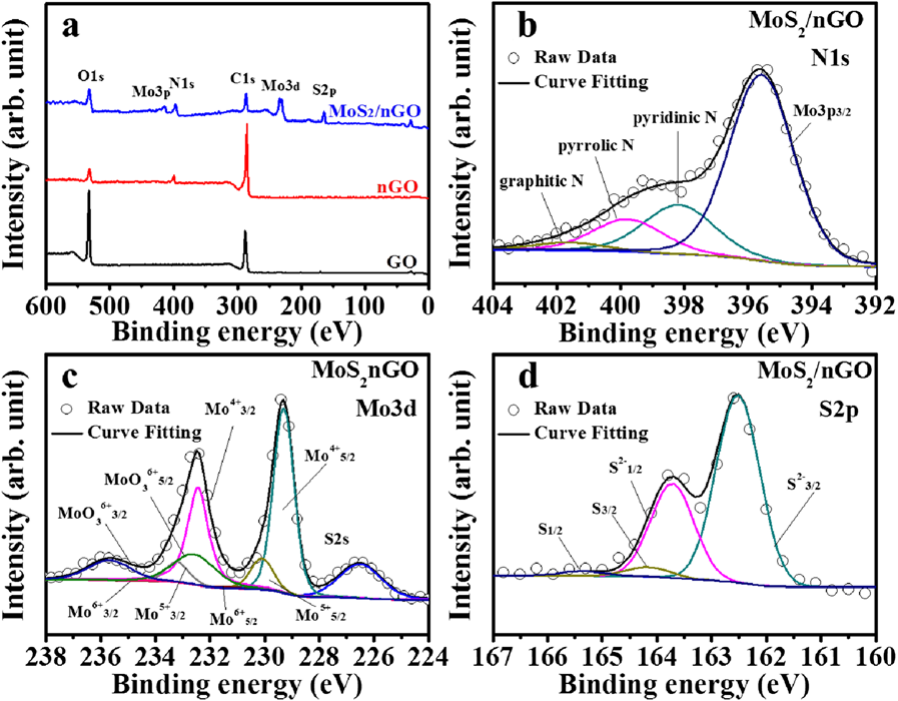
**a** XPS survey spectra and high-resolution XPS analysis of (**b**) N1s, (**c**) Mo3d, and (**d**) S2p

XPS was applied to analyze the chemical states of the GO, nGO, and MoS_2_/nGO nanocomposites. The curve fitting was performed by using a Gaussian–Lorentzian peak after the Shirley background correction. Figure 1 and Table 1 show the XPS fitting results of various CEs. Figure 1a shows the wide spectral region and illustrates the differences in the relative intensities of the characteristic peaks of the elements present. As we can see from Fig. 1a, compared with GO, the N1s of nGO can be found, and the C1s intensity of nGO dramatically decreased after the nitrogen-doped process. The decline O1s peak of nGO indicated that lots of oxygen group defects on the GO surface were repaired. Figure 1b shows the high-resolution N1s spectra of the MoS_2_/nGO nanocomposite; three N configurations with graphitic N (401.6 eV), pyridinic N (398.4 eV), and pyrrolic N (400 eV) were presented [20, 32–34]. The MoS_2_ nanosheets were synthesized onto the surface of the nanosheet-like nGO as a MoS_2_-nGO nanocomposite material by an easy thermal reduction method.

**Table 1:** N1s, Mo3d, and S2p peak positions and atomic percentages of MoS_2_/nGO

Peak	Fitting of the peak binding energy (eV) (atomic percentage (%))							
	Pyridinic *N*		Pyrrolic *N*			Graphitic *N*		
N1s	398.4 (54.89)		400.0 (36.26)			401.6 (8.83)		
	Mo^4+^3d_5/2_	Mo^4+^3d_3/2_	Mo^5+^3d_5/2_	Mo^5+^3d_3/2_	Mo^6+^3d_5/2_	Mo^6+^3d_3/2_	Mo^6+^3d_5/2_	Mo^6+^3d_5/2_
Mo3d	229.3 (38.87)	231.4 (25.91)	230.1 (7.34)	233.2 (4.89)	231.3 (0.27)	234.4 (0.18)	232.5 (13.50)	235.6 (9.00)
	S^2−^2p_3/2_		S^2−^2p_1/2_		S_2_^2−^2p_3/2_		S_2_^2−^2p_1/2_	
S2p	162.0 (63.50)		163.2 (31.75)		163.3 (3.16)		164.5 (1.58)	

Figure 1c shows the high-resolution Mo3d spectra of the MoS_2_/nGO nanocomposite, the 3d_5/2_ and 3d_3/2_ of the four valence states of Mo: Mo^4+^_3d5/2_ (229.3 eV) and Mo^4+^ 3d_3/2_ (232.4 eV); Mo^5+^ 3d_5/2_ (230.1 eV) and Mo^5+^ 3d_3/2_ (233.2 eV); Mo^6+^ 3d_5/2_ (231.3 eV) and Mo^6+^ 3d_3/2_ (234.4 eV); and Mo^6+^_3d5/2_ (232.5 eV) and Mo^6+^ 3d_3/2_ (235.6 eV), can be assigned to MoS_2_, Mo_2_S_5_, MoS_3_, and MoO_3_, respectively [35]. Figure 1d shows that high-resolution S2p spectra of the MoS_2_/nGO nanocomposite, the 2p_3/2_ and 2p_1/2_, of the two valence states of S were assigned to MoS_2_ and Mo_2_S_5_, respectively. This phenomenon might correspond to the MoS_3_ with a formula of [Mo(4^+^)(S_2_)^2−^S_2_^−^] and the intermediate product Mo_2_S_5_ [35]. These binding energies can be attributed to the MoS_2_ crystal as previously reported [24, 36]. The summary of the peak positions (N1s, Mo3d, and S2p) and the atomic percentages were also given in Table 1.

On the other hand, the molybdenum sulfide precursor followed by the thermal reduction method was to make the sulfidation process to synthesize the crystallized MoS_2_ based on the following reaction (Eq. (1) and Eq. (2)) [37]:

1 $$ {\left({\mathrm{NH}}_4\right)}_2{\mathrm{MoS}}_4\to\ 2{\mathrm{NH}}_3 + {\mathrm{H}}_2\mathrm{S} + {\mathrm{MoS}}_3 $$

2 $$ {\mathrm{MoS}}_3 + \mathrm{H}\ \to\ {\mathrm{MoS}}_2 + {\mathrm{H}}_2\mathrm{S} $$

**Fig. 2:**
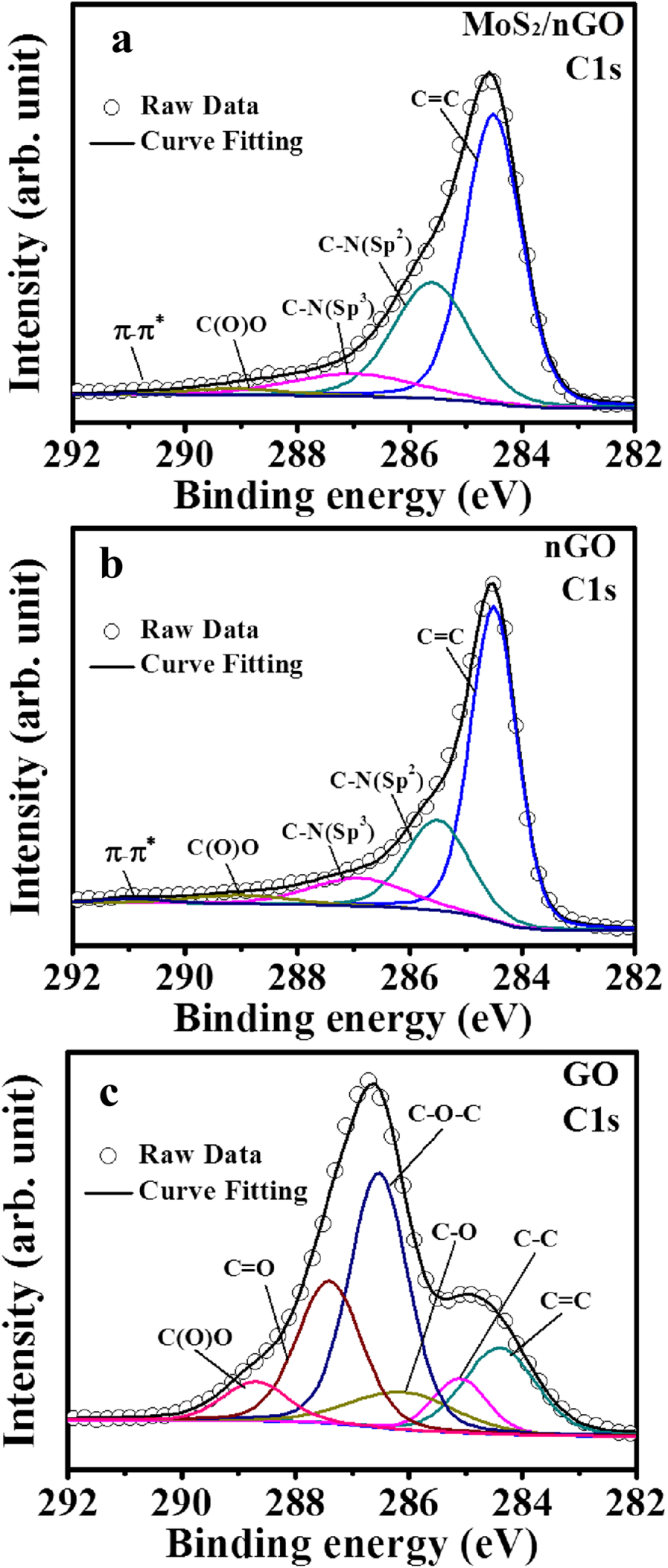
C1s peak in the XPS spectra of (**a**) MoS2/nGO, (**b**) nGO, and (**c**) GO

Furthermore, in order to intensively study the chemical states of nGO, we also paid the attention to analyze the C1s peaks as shown in Fig. 2. Figure 2 and Table 2 show the C1s fitting results of various CEs. There were five peaks that can be obtained from the MoS_2_/nGO and nGO in Fig. 2a, b, respectively, which were the C=C (284.5 eV), C–N(sp2) (285.5 eV), C–N(sp3) (287.0 eV), C(O)O (289.1 eV), and the π–π* (291 eV), respectively. There were six peaks that can be obtained from GO in Fig. 2c, which were the C=C (284.4 eV), C–C (285.1 eV), C–O (286.1 eV), C–O–C (286.5 eV), C=O (287.4 eV), and C(O)O (288.7 eV), respectively [38]. The summary of the peak positions and the atomic percentages is also given in Table 2. As we can see from Fig. 2c, the C1s core level spectra C–O–C of GO indicated a high degree of oxidation. However, after the nitrogen-doped process, the C–O–C peaks in both MoS_2_/nGO (Fig. 2a) and nGO (Fig. 2b) almost disappeared and the C–N groups (C–N(sp2) and C–N(sp3)) were obtained obviously [33]. According to this change, this phenomenon indicated that the oxygen atoms were almost replaced by nitrogen atoms. The results confirmed that nitrogen atoms were introduced into the GO structure, which improved the conductivity of the basal plane of nGO.

**Table 2:** C1s peak positions and atomic percentages of GO, nGO, and MoS2/nGO

Samples	Fitting of the C1s peak binding energy (eV, atomic percentage (%))					
	C=C	C–N(sp2)	C–N(sp3)	C(O)O		π–π*
MoS_2_/nGO	284.5 (54.94)	285.5 (30.05)	287.0 (10.18)	289.1 (2.52)		291 (2.28)
nGO	284.6 (57.17)	285.6 (22.63)	287.0 (13.19)	289.1 (3.85)		291 (3.14)
	C=C	C–C	C–O	C–O–C	C=O	C(O)O
GO	284.4 (15.73)	285.1 (7.42)	286.1 (9.81)	286.5 (37.80)	287.4 (22.95)	288.7 (6.26)

**Fig. 3:**
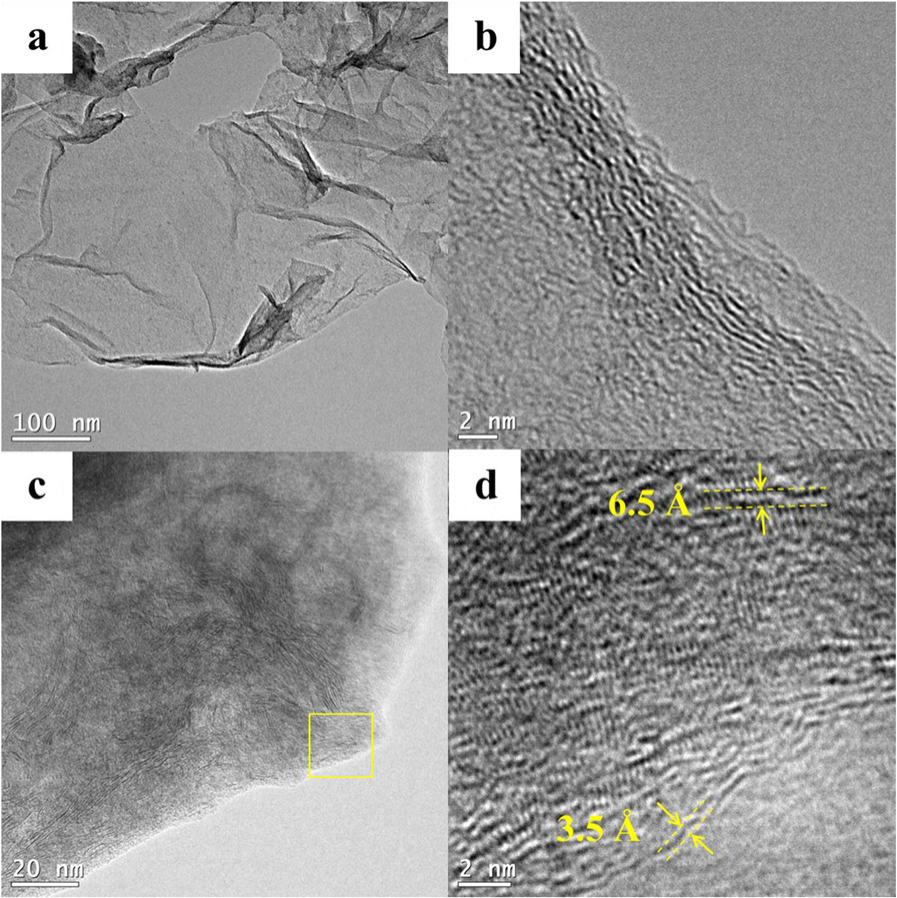
**a** TEM and (**b**) HRTEM images of nGO and (**c**) TEM and (**d**) HRTEM images of MoS_2_/nGO

Figure 3a, b shows the TEM and HRTEM images of nGO nanosheets, respectively. Figure 3c shows the TEM images of the MoS_2_/nGO nanocomposite; the nGO served as the template where MoS_2_ was anchored. The nanostructure of MoS_2_/nGO was investigated by HRTEM (as shown in Fig. 3d); the sheet-like MoS_2_ was successfully formed onto the nGO surface. As we can see from Fig. 3c, there were many interlayers of the MoS_2_ chemically reduced on the nGO surface. The edge planes of MoS_2_ provided plenty active sites to enhance the electrocatalytic activities. Figure 3d shows the HRTEM of the interlayer distance of the MoS_2_ which was about 6.5 Å [26], corresponding to the spacing between (002) planes of MoS_2_ [39]. Furthermore, the interlayer distance of the nGO was about 3.5 Å [40], which was larger than that of graphene nanosheets of 3.35 Å; it was probably due to nGO which still remained as an oxygen-containing functional group [41].

**Fig. 4:**
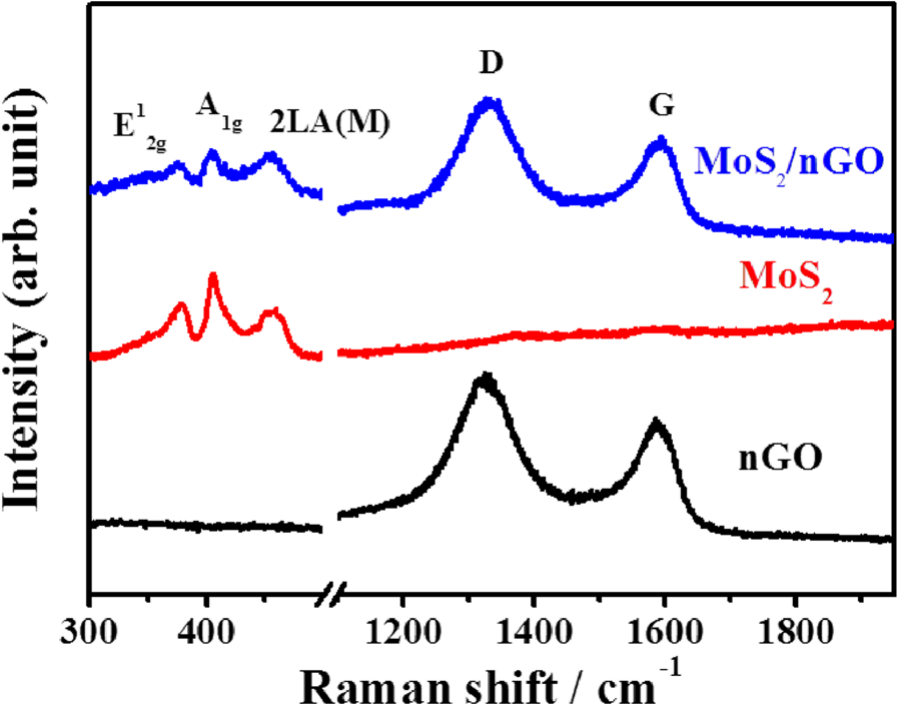
Raman spectrum of the nGO, MoS_2_, and MoS_2_/nGO. The *D*, *G*, *E* ^*1*^_*2g*_, *A*_*1g*_, and *2LA(M)* modes were marked in the MoS_2_/nGO spectrum

Figure 4 shows the Raman spectrum of nGO, MoS_2_, and MoS_2_/nGO. The nGO exhibited two graphitic characteristic peaks at 1354.6 and 1597.6 cm^−1^, corresponding to D and G bands, respectively [42]. G band corresponded to the in-plane bond stretching of pairs of graphitic sp2 carbon atoms; D band corresponded to the defects of lattice and lattice distortion. The three MoS_2_ characteristic peaks at 378.9, 404.3, and 453.8 cm^−1^ corresponded to E^1^_2g_, A_1g_, and 2LA(M) modes, respectively [43]. The E^1^_2g_ mode was associated with the opposite vibration of two S atoms with respect to the Mo atom. The A_1g_ mode was associated with the out-of-plane vibration of only S atoms in opposite directions. The 2LA(M) was attributed to the resonance second-order Raman effects that appear when the excitation wavelength of the 632.8 nm laser was near that of the MoS_2_ electronic absorption bands [43].

### Electrocatalytic Properties

**Fig. 5:**
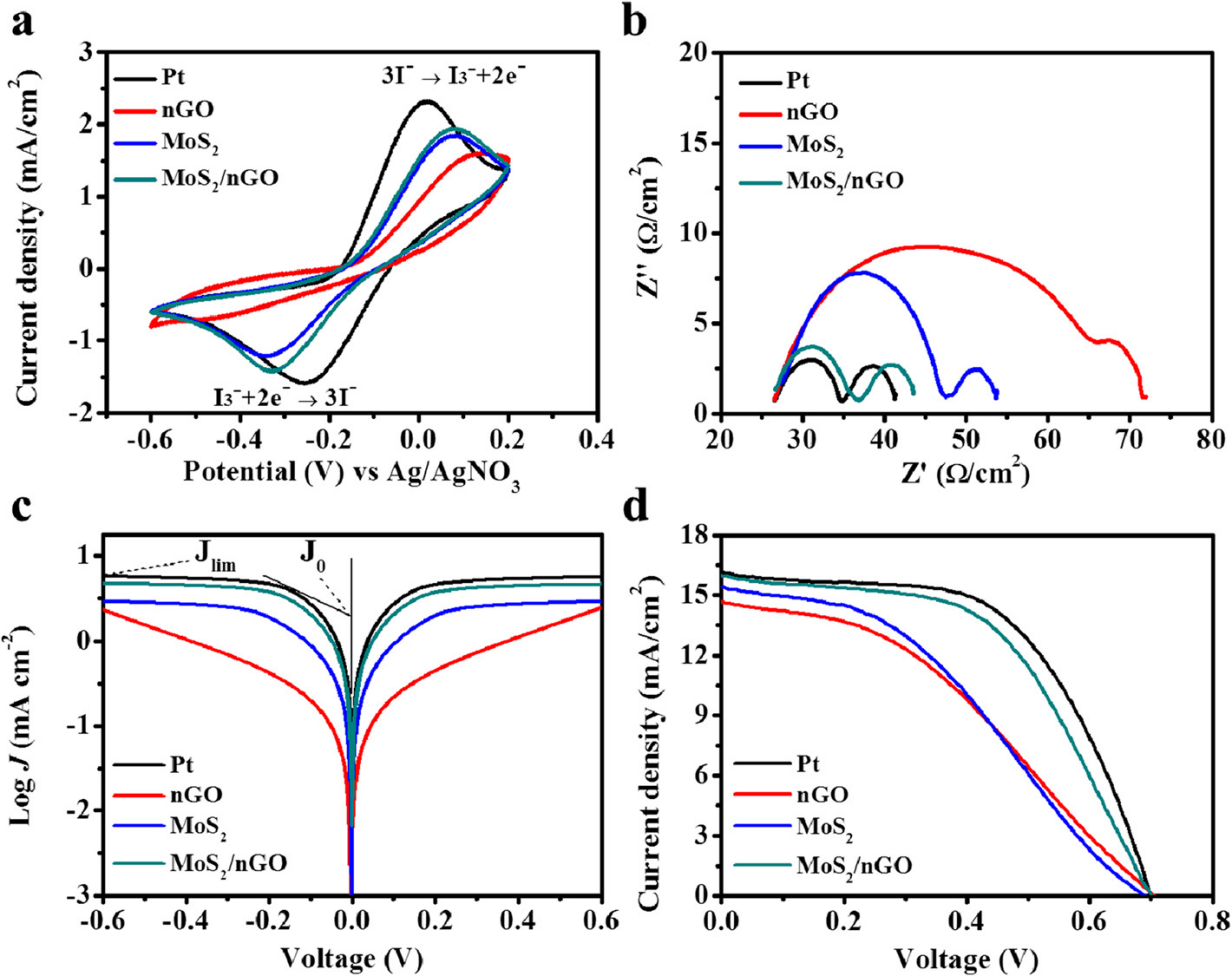
**a** CV results of the I^−^/I_3_^−^ redox system for *Pt*, *nGO*, *MoS*_*2*_, and *MoS*_*2*_ */nGO* CEs. **b** Nyquist plots based on *Pt*, *nGO*, *MoS*_*2*_, and *MoS*_*2*_ */nGO* CEs. **c** Tafel polarization curves of the *Pt*, *nGO*, *MoS*_*2*_, and *MoS*_*2*_ */nGO* CEs. **d** Photovoltaic characteristic efficiencies of DSSCs based on *Pt*, *nGO*, *MoS*_*2*_, and *MoS*_*2*_ */nGO* CEs

To investigate the electrocatalytic properties of Pt, MoS_2_, nGO, and MoS_2_/nGO toward the reduction of I_3_^−^, the CV analysis was carried out with a scan rate of 50 mV/s. As shown in Fig. 5a, the redox couple peaks corresponded to the reduction of I_3_^−^ (cathodic peak current (*I*_pc_) : I_3_^−^ + 2e^−^ → 3I^−^) and the oxidation of I^−^ (anodic peak current (*I*_pa_) : 3I^−^ → I_3_^−^ + 2e^−^) in the CV measurement [44]. The *I*_pc_ value of MoS_2_/nGO (−1.41 mA/cm^2^) was stronger than those of MoS_2_ (−1.21 mA/cm^2^) and nGO (−0.69 mA/cm^2^). It indicated that MoS_2_ CE could enhance the charge transport rate. The electrocatalytic activity and the redox barrier of I_3_^−^/I^−^ couples could be evaluated in terms of its cathodic peak current (*I*_pc_) and the peak to peak voltage separation (*E*_pp_), respectively. The *E*_pp_ of the MoS_2_/nGO CE became relatively lower when compared to that of MoS_2_ and nGO. This was immediately responsible for the lower overpotential losses in the MoS_2_/nGO CE than those of the MoS_2_ and nGO CEs [44]. It also signified that dispersing intrinsically electrocatalytic MoS_2_ onto the nGO surface led to enhance the electrocatalytic activity for I_3_^−^ reduction. EIS analysis was used to examine the electron interface transfer activity. Figure 5b shows the Nyquist plots of various CEs: the intercept on the horizontal axis was the series resistance (*R*_s_); the left semicircle at high frequency represented the charge transfer resistance (*R*_ct_) at electrode/electrolyte interface; and the right semicircle at low frequency represented the Nernst diffusion impedance (*N*_diff_) in the electrolyte [44]. The *R*_ct_ values of the MoS_2_/nGO, MoS_2_, and nGO were 5.4 Ω/cm^2^, 10.1 Ω/cm^2^, and 21.3 Ω/cm^2^, respectively. The EIS results showed that the *R*_ct_ of MoS_2_/nGO CE was smaller than that of nGO and MoS_2_ CEs. The *N*_diff_ values of the MoS_2_/nGO, MoS_2_, and nGO were 8.3 Ω/cm^2^, 7.1 Ω/cm^2^, and 8.1 Ω/cm^2^, respectively. The similar *N*_diff_ values of various CEs due to the same thickness spacers were used for the EIS measurements. To further confirm the electrocatalytic activity of the prepared CEs, the Tafel polarization measurements were also carried out to examine the exchange current density (*J*_0_) and the limiting current density (*J*_lim_). Figure 5c shows the Tafel polarization measurements of various CEs; the *J*_0_ was related to the charge transfer activity in the interface between CE and electrolyte, which can be estimated from the extrapolated intercepts of the anodic and cathodic branches of the corresponding Tafel curves [45]. The *J*_lim_ depended on the diffusion coefficient and concentration of the I^−^/I_3_^−^ redox couple. *J*_0_ and *J*_lim_ were related to the following reaction [9, 22]:

3 $$ {J}_0=\frac{RT}{n\mathrm{F}{R}_{\mathrm{ct}}} $$

4 $$ D=\frac{1}{2n\mathrm{F}C}{J}_{\lim } $$

where *R* is the gas constant, *T* is the temperature, F is the Faraday constant, *l* is the spacer thickness, *C* is the concentration of I_3_^−^ species, and *n* represents the number of electrons involve in the reaction at the electrode/electrolyte interface.

The *J*_0_ varies inversely with *R*_ct_ as shown in Eq. (3); the variation of *J*_0_ obtained from the Tafel curve is generally in accordance with the change tendency of *R*_ct_ obtained from the EIS plot. In addition, the intersection of the cathodic branch and the equilibrium potential line in the Tafel measurement can be considered as the *J*_lim_. As shown in Fig. 5c, the *J*_0_ and *J*_lim_ of MoS_2_ CE (1.32 and 2.88 mA/cm^2^) were higher than those of nGO CE (0.14 and 2.29 mA/cm^2^). The results indicated that the charge transfer abilities of MoS_2_ were much better than those of nGO. Compared with the MoS_2_ and nGO CEs, the MoS_2_/nGO nanocomposite CE showed the promoted *J*_0_ and *J*_lim_ values of 2.64 and 4.67 mA/cm^2^, respectively. MoS_2_/nGO nanocomposite showed the highest *J*_0_ and *J*_lim_ and the lowest *R*_ct_. The result indicated that MoS_2_/nGO CE provided the outstanding electron transfer ability in the electrode–electrolyte interface. Additionally, MoS_2_/nGO CE showed the enhanced *J*_lim_ value, which indicated a faster diffusion rate of the I^−^/I_3_^−^ redox couples; this phenomenon was related to the better diffusion coefficient in Eq. (4). According to the results of CV, EIS, and the Tafel polarization, the MoS_2_/nGO CE demonstrated the superior electron transfer performance at the electrode–electrolyte interface and provided a lower energy barrier for the redox reactions. The results obtained from CV, EIS, and the Tafel polarization were summarized in Table 3.

**Table 3:** Photovoltaic parameters and the electrochemical parameters from EIS, CV, and Tafel polarization measurements based on various CEs

	Pt	nGO	MoS_2_	MoS_2_/nGO
*I*_pc_ (mA/cm^2^)	−1.57	−0.69	−1.21	−1.41
*E*_pp_ (V)	0.26	0.61	0.43	0.40
*R*_s_ (Ω/cm^2^)	26.3	25.7	26.0	25.7
*R*_ct_ (Ω/cm^2^)	4.3	21.3	10.1	5.4
*N*_diff_ (Ω/cm^2^)	7.6	8.1	7.1	8.3
*J*_0_ (mA/cm^2^)	3.19	0.14	1.32	2.64
*J*_lim_ (mA/cm^2^)	5.65	2.29	2.88	4.67
*V*_oc_ (V)	0.70	0.71	0.69	0.70
*J*_sc_ (mA/cm^2^)	16.14	14.66	15.39	15.98
F.F.	0.57	0.38	0.39	0.53
*η* (%)	6.43	3.95	4.09	5.95

### Photovoltaic Performance of DSSCs

In order to investigate the photovoltaic performance of DSSCs, the cell performance were characterized by short-circuit current density (*J*_sc_), open-circuit voltage (*V*_oc_), fill factor (F.F.), and PCE (η (%)). The fill factor and the PCE of the DSSCs could be estimated from Eq. (5) and Eq. (6) [46]:

5 $$ \mathrm{F}.\mathrm{F}.=\frac{V_{\max } \times {J}_{\max }}{V_{\mathrm{oc}} \times {J}_{\mathrm{sc}}} $$

6 $$ \eta \left(\%\right)=\frac{V_{\mathrm{oc}} \times {J}_{\mathrm{sc}} \times \mathrm{F}.\mathrm{F}.}{P_{\mathrm{in}}}\times 100 $$

where *P*_in_ is the power input and *V*_max_ and *J*_max_ are the voltage and the current density for the maximum power output, respectively.

The photocurrent–voltage characteristics of DSSCs with various CEs including Pt, nGO, MoS_2_, and MoS_2_/nGO were shown in Fig. 5d. The corresponding photovoltaic parameters were also summarized in Table 1. From the photovoltaic characteristics, the DSSCs with the nGO CE showed the lowest *J*_sc_ (14.66 mA/cm^2^) and F.F. (0.38) and exhibited a lower PCE (3.95 %). The corresponding photovoltaic parameters of MoS_2_ CE showed that the PCE (4.09 %), *J*_sc_ (15.39 mA/cm^2^), and F.F. (0.39) were higher than those of nGO CE. The results showed that the catalytic ability of MoS_2_ was higher than that of nGO. The *J*_sc_ and F.F. values of the MoS_2_ CE were better than those of nGO CE, which might be due to the plenty edge sites of the MoS_2_. Compared with the nGO and MoS_2_, MoS_2_/nGO showed the excellent *J*_sc_ (15.98 mA/cm^2^) and F.F. (0.53) and resulted in the outstanding PCE (5.95 %). In addition, all the CE materials exhibited the similar *V*_oc_ values, because the DSSC devices in this study used the same WE and electrolyte. In summary, the 2D nanocomposite of MoS_2_/nGO combined the advantages of nGO and MoS_2_. The nGO provided a large surface area to anchor MoS_2_, and the plenty edge sites of the anchored MoS_2_ promoted the electrocatalytic activities. Furthermore, the promoted values of *J*_sc_ and F.F. made the PCE (5.95 %) of MoS_2_/nGO nanocomposite CE comparable to the conventional Pt CE (6.43 %).

## Conclusions

Based on the XPS, Raman spectrum, and HRTEM results, the sheet-like MoS_2_ was confirmed to form onto the surface of nGO nanosheet as the 2D nanocomposite of MoS_2_/nGO. According to CV, EIS, and the Tafel analyses, MoS_2_/nGO owned the outstanding electrocatalytic activities. The MoS_2_/nGO combined the advantages of the high specific surface of nGO and the plenty edge sites of MoS_2_ and showed the properties different from those of their individual constituents to create a new outstanding property. Finally, the DSSCs assembled with MoS_2_/nGO CE exhibited excellent photovoltaic conversion efficiency (5.95 %) which was comparable to the DSSC with the conventional Pt CE (6.43 %). This work demonstrated that the MoS_2_/nGO nanocomposite could offer a low-cost alternative to replace the expensive Pt in DSSCs.
